# Management of complex renal stones in horseshoe kidney using real-time intrarenal pressure monitoring with artificial intelligence in retrograde intrarenal surgery: A case report

**DOI:** 10.1016/j.eucr.2026.103366

**Published:** 2026-02-03

**Authors:** Armand Achmadsyah, Favian Ariiq Rahmat, Nadhif Faza Ananda, Mukhlis Akmal Taher, Gerhard Reinaldi Situmorang, Widi Atmoko, Nur Rasyid, Ponco Birowo

**Affiliations:** a Department of Urology, Faculty of Medicine, Universitas Indonesia, Dr. Cipto Mangunkusumo Hospital, Jakarta, Indonesia; b Faculty of Medicine, Universitas Indonesia, Jakarta, Indonesia

**Keywords:** Intrarenal pressure, Artificial intelligence, retrograde intrarenal surgery, Horseshoe kidney, Complex renal stones

## Abstract

Retrograde intrarenal surgery (RIRS) in horseshoe kidneys is technically challenging due to altered anatomy and unstable intrarenal pressure (IRP). We report a case of a 45-year-old male with complex renal stones in a horseshoe kidney managed using artificial intelligence (AI)-assisted real-time IRP monitoring during RIRS. The AI system dynamically regulated irrigation and suction to maintain IRP within a safe range (∼30 mmHg), improving intraoperative visibility and procedural control. Stone clearance was achieved without complications. This case highlights the feasibility and potential safety benefits of AI-assisted IRP monitoring in anatomically complex kidneys.

## Highlights

• First documented case applying AI-assisted intrarenal pressure (IRP) monitoring in retrograde intrarenal surgery (RIRS) for a horseshoe kidney.
• The AI-driven system maintained stable intrarenal pressure (∼30 mmHg) and improved intraoperative visibility throughout the procedure.
• This case demonstrates the potential of AI-assisted IRP monitoring to enhance procedural safety in anatomically complex kidneys.

## 1 Introduction

Intrarenal pressure (IRP) management is a cornerstone of retrograde intrarenal surgery (RIRS), ensuring both patient safety and procedural success. Elevated IRP can lead to complications such as pyelovenous backflow, infection, and sepsis, while low IRP compromises visibility, prolonging operative time and reducing efficacy.^1^^,^^2^ Maintaining IRP below 40 cm H_2_O—the threshold considered safe to minimize these risks—is particularly challenging in cases with anatomical variations like horseshoe kidneys, where altered renal structures further complicate surgical access.^1^

In these complex scenarios, precise control of IRP becomes even more critical. Traditional methods of IRP monitoring and manual irrigation adjustments often fall short in balancing field clarity and safety, especially in anatomically challenging cases. This gap has spurred the development of advanced technologies, such as artificial intelligence (AI), which offers real-time monitoring and automated adjustments to maintain IRP within an optimal range. By dynamically regulating irrigation flow rates, AI ensures surgical field visibility while mitigating risks associated with fluctuating IRP.^3^

Despite its potential, the application of AI-assisted IRP control in managing complex cases like horseshoe kidneys has not been fully explored. This case report presents the first documented use of AI in RIRS for a horseshoe kidney, highlighting its feasibility and potential benefits in addressing the dual challenges of complex renal stones and anatomical abnormalities. This case report has been prepared following the SCARE 2025 criteria.

## 2 Case presentation

A 45-year-old male presented with a three-month history of intermittent dull pain in the left flank, rated 2–3 on the Visual Analog Scale (VAS). The pain was not accompanied by fever, hematuria, or urinary symptoms. The patient denied any history of trauma, systemic illnesses, or similar conditions in the family. He reported a daily fluid intake of approximately 1000–1500 mL, with a urinary output of 1500–2000 mL, which was yellow and clear. The patient had a history of tuberculosis and was in the third month of intensive-phase antituberculosis treatment. Previous surgical interventions included the placement of a left double-J (DJ) stent in May 2024 due to obstructive uropathy caused by multiple left renal stones.

**Fig. 1:**
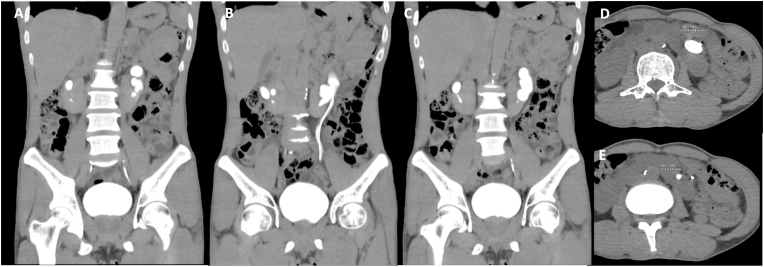
Preoperative imaging findings in a patient with a horseshoe kidney and complex renal stones. **(A**–**C)** Coronal contrast-enhanced CT urography images demonstrating a horseshoe kidney with fused lower poles and multiple left renal stones, including the largest stone measuring 21.9 × 18.2 × 13.2 mm. **(D**–**E)** Axial images confirming high-density renal stones (1297 HU) within the left collecting system.

On physical examination, the patient was hemodynamically stable with normal abdominal findings. His body mass index was 18.9 kg/m^2^. There were no signs of flank tenderness, masses, or bulging. Preoperative laboratory investigations revealed a hemoglobin level of 11 g/dL, hematocrit of 33.6%, leukocyte count of 10,260/μL, and platelet count of 415,000/μL. Renal function tests showed a ureum level of 23.5 mg/dL and creatinine of 0.9 mg/dL. Serum electrolytes, including sodium (135 mEq/L), potassium (3.7 mEq/L), and chloride (105.1 mEq/L), were within normal limits. A contrast-enhanced CT urogram (Fig. 1A–E) revealed a horseshoe kidney with fused lower poles, multiple left renal stones, the largest measuring 21.9 x 18.2 × 13.2 mm with a Hounsfield Unit (HU) of 1297, and grade 2 hydronephrosis. The right kidney and urinary bladder appeared normal.

**Fig. 2:**
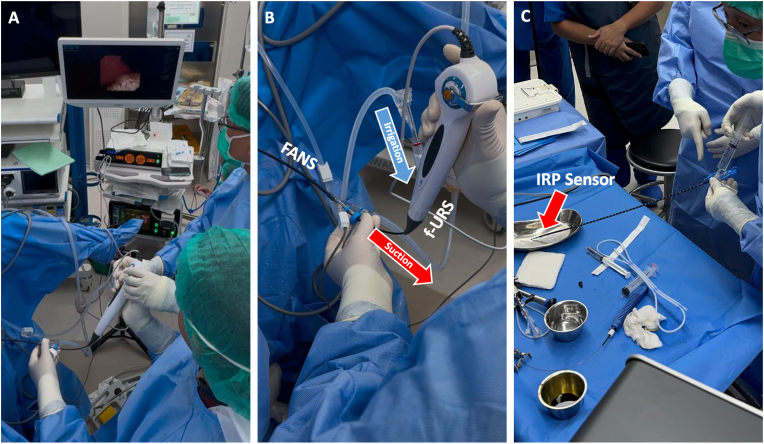
Intraoperative setup and function of AI-assisted IRP monitoring during RIRS. (**A**) Intraoperative view during the operation. (**B**) Mechanism of irrigation and suction using the AI-assisted IRP monitoring system. (**C**) IRP sensor located at the tip of the FANS system for real-time pressure monitoring.

**Fig. 3:**
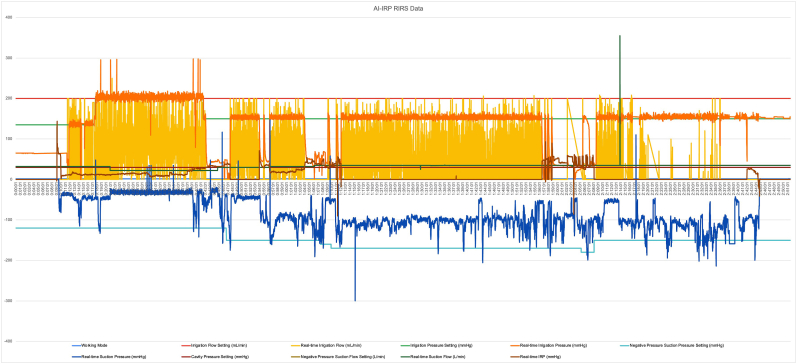
Data from the AI-assisted IRP monitoring system during RIRS, showing irrigation flow, pressure, suction settings, and real-time intrarenal pressure adjustments.

The patient was diagnosed with grade 2 hydronephrosis caused by multiple left renal stones in a horseshoe kidney. The planned treatment was retrograde intrarenal surgery (RIRS) with artificial intelligence (AI)-assisted intrarenal pressure (IRP) monitoring (Fig. 2). The procedure utilized an i-MIMER Intelligent IRP-Controlled Irrigation and Suction Pump (Dongguan ZSR Biomedical Technology, Guangdong, China) connected to a ZSR IRP-Sheath for real-time IRP control. During the surgery, performed under general anesthesia with the patient in the lithotomy position, initial challenges were encountered with the AI system in stabilizing IRP. The preset suction and irrigation parameters were configured for anatomically normal kidneys, which proved suboptimal for the altered anatomy of the horseshoe kidney. Adjustments were made to the settings, including an irrigation flow of 200 mL/min, irrigation pressure of 135 mmHg, suction flow of 32 L/min, suction pressure of −120 mmHg, and a cavity pressure of 30 mmHg. Following these modifications, the IRP stabilized, enabling the procedure to continue effectively (Fig. 3)

**Fig. 4:**
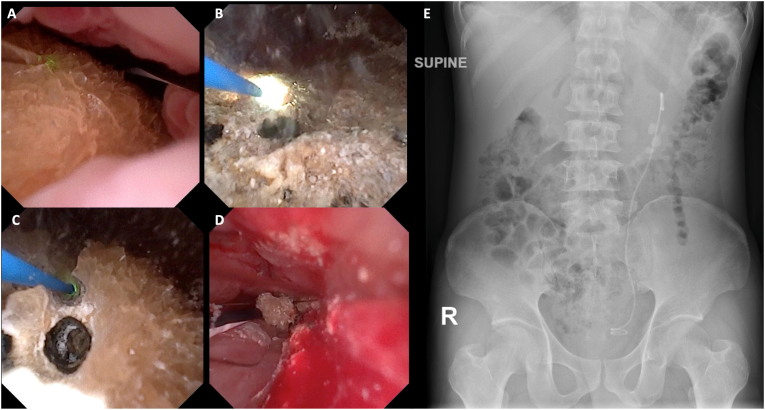
Intraoperative findings and postoperative outcome of RIRS in a horseshoe kidney. (**A**) Initial visualization of the renal stone before laser lithotripsy. (**B-C**) The progression of laser lithotripsy, showing the fragmentation of the stone. (**D**) Final intraoperative view showing the last remaining stone fragments before evacuation. **(E)** Postoperative plain abdominal radiograph (supine position) demonstrating correct placement of the left double-J stent with no visible residual radiopaque stones.

A 7.5 Fr single-use digital flexible ureteroscope (ZSR, Dongguan ZSR Biomedical Technology, Guangdong, China) (Fig. 4A–D) was introduced through a 10/12 Fr ZSR IRP-Sheath (ZSR, Dongguan ZSR Biomedical Technology, Guangdong, China) under fluoroscopic guidance. The sheath was then connected to the AI-IRP monitoring system to enable real-time pressure regulation and balanced irrigation–suction control. Retrograde pyelography confirmed the presence of filling defects in the left renal pelvis. Laser lithotripsy was performed using a Fiber Dust Thulium Fiber Laser (Quanta System, Samarate, Italy) in both dusting and fragmentation modes with a 200-μm fiber. In dusting mode, the laser was set to 20 W (0.2 J, 100 Hz), while fragmentation mode utilized a setting of 22.5 W (1.5 J, 15 Hz). Stone fragments were extracted using a combination of suction and a stone basket. Following comprehensive dusting and suction, intraoperative evaluation showed no visible stone fragments. Active bleeding and laser-induced injuries to the infundibulum were not observed. A 4.7 Fr DJ stent was inserted into the left ureter to ensure adequate postoperative drainage.

The total operative time was 150 minutes with an estimated blood loss of approximately 5 mL. Postoperatively, the patient was hemodynamically stable and reported significant relief from symptoms. The postoperative pain was mild (VAS 2/10) and controlled with non-opioid analgesics. Laboratory results revealed a hemoglobin level of 13 g/dL, hematocrit of 39.4%, leukocyte count of 9800/μL, and platelet count of 307,000/μL. Serum electrolytes, including sodium (135 mEq/L), potassium (4.3 mEq/L), and chloride (102.6 mEq/L), remained within normal limits. A plain abdominal radiograph (BNO) (Fig. 4E) obtained postoperatively showed no visible residual stones. The double-J stent was seen in good position, and the patient was discharged on postoperative day three with satisfactory recovery. Follow-up was planned in the urology outpatient clinic to monitor clinical progress. The patient was satisfied with the treatment and recovery.

## 3 Discussion

This case highlights the successful management of a complex renal stone in a horseshoe kidney using retrograde intrarenal surgery (RIRS) with artificial intelligence (AI)-assisted intrarenal pressure (IRP) monitoring. Horseshoe kidneys present distinctive anatomical and technical challenges in endourology due to renal malrotation, high ureteral insertion, and a flattened renal pelvis that complicate surgical access and impair pelvic drainage. These anatomical variations predispose patients to elevated intrarenal pressure and infection during irrigation, often resulting in prolonged operative times and lower stone-free rates compared with anatomically normal kidneys. Flexible ureteroscopy has been shown to achieve higher stone clearance and fewer complications than extracorporeal shock wave lithotripsy in horseshoe kidneys because of its ability to directly access and fragment stones under visual control.^1^^,^^4^ However, persistent challenges related to intraoperative visibility and pressure stability remain, underscoring the need for advanced technologies capable of maintaining a controlled intrarenal environment. The integration of AI-assisted IRP monitoring in this case addressed these limitations by dynamically maintaining stable intrarenal pressure and optimizing procedural outcomes.

Intrarenal pressure is a key factor influencing the safety and efficacy of RIRS. Elevated IRP, exceeding 40 cm H_2_O, can cause pyelovenous backflow, increasing the risk of infection, systemic inflammatory response syndrome, and sepsis. Conversely, low IRP compromises the surgical field, reducing visibility and procedural efficiency.^1^^,^^5^

Maintaining IRP within an optimal range (∼30 mmHg ± 5) is crucial to balancing these risks, especially in cases with anatomical abnormalities such as horseshoe kidneys.^1^ Despite its importance, traditional methods for IRP monitoring, such as ureteral catheters, sensor wires, and proximal pressure sensors, face significant limitations, including detection lag, indirect measurements, and lack of real-time integration into irrigation systems.^6^ These challenges have necessitated the development of innovative tools, such as AI-assisted systems, to provide continuous and precise IRP control during RIRS.

**Fig. 5:**
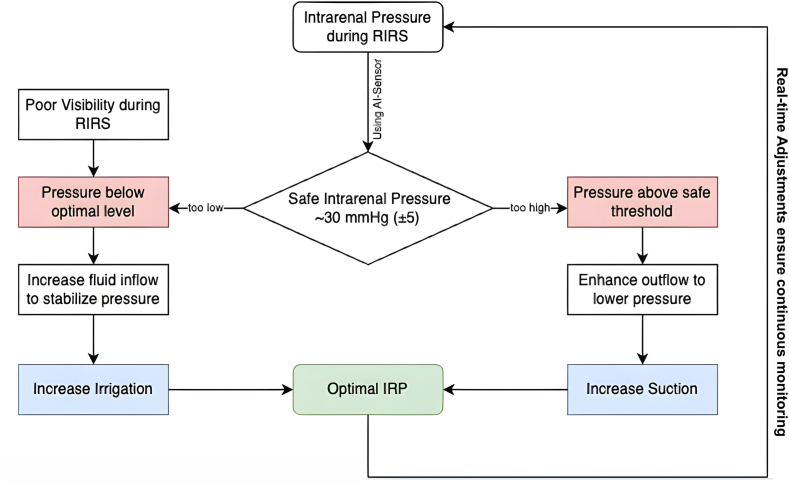
Mechanism of AI-assisted intrarenal pressure (IRP) monitoring during RIRS.

The AI-assisted IRP monitoring system used in this case dynamically adjusted irrigation and suction parameters in real time, ensuring stable IRP throughout the procedure (Fig. 5). This automation reduced the need for manual adjustments, allowing the surgeon to focus entirely on stone treatment. A study by Zhu et al. (2025) demonstrated that an intelligent IRP-controlled suction ureteral access sheath could effectively maintain intrarenal pressure within a safe range of 25–35 mmHg, even during high-flow irrigation, while simultaneously shortening operative time by approximately 25% compared with conventional systems. The study also reported comparable stone-free rates (89.7% vs 87.5%) and a lower incidence of postoperative infection (0% vs 4%) in the IRP-monitored group.^7^ Moreover, the system's ability to adapt to anatomical variations, such as those seen in horseshoe kidneys, demonstrated its potential for broader clinical applications. As illustrated in Fig. 3, the system continuously monitored IRP, increasing irrigation flow when pressure dropped below the optimal range and enhancing suction when pressure exceeded the safe threshold.

In addition to regulating IRP, these systems have the potential to indirectly influence intrarenal temperature (IRT), an emerging consideration in endourology. High IRT during laser lithotripsy, resulting from prolonged irrigation flow, can lead to thermal injury of renal tissues.^2^^,^^8^ By optimizing irrigation and suction, AI-assisted systems help maintain a safe IRT, reducing the risk of thermal complications.

Standard RIRS relies on manual adjustments for irrigation and suction, which can result in fluctuating IRP levels and increased cognitive demands on the surgeon. Although the use of AI in IRP monitoring is still emerging, its ability to automate these adjustments represents a significant step forward in optimizing procedural safety and efficiency. Initial challenges with IRP stabilization in this case highlighted the need for AI systems to account for anatomical anomalies. The pre-set parameters, optimized for anatomically normal kidneys, were insufficient for the altered anatomy of a horseshoe kidney. Adjustments to irrigation flow (200 mL/min), irrigation pressure (135 mmHg), suction flow (32 L/min), suction pressure (−120 mmHg), and cavity pressure (30 mmHg) were necessary to achieve stable IRP.^3^^,^^9^ These modifications underscore the importance of adaptable algorithms in expanding the utility of AI-assisted systems in diverse clinical scenarios.

This case demonstrates the feasibility and potential benefits of AI-assisted IRP monitoring in managing complex renal stones, particularly in anatomically challenging cases. While promising, this report highlights the need for further research to validate these findings in larger cohorts. Future developments should focus on refining AI algorithms to better accommodate anatomical variations and exploring the cost-effectiveness of integrating AI systems into routine endourological practice. Additionally, the integration of real-time IRT monitoring into AI systems could further enhance procedural safety, especially during laser lithotripsy.

## 4 Conclusion

The integration of AI-assisted IRP monitoring in RIRS represents a significant advancement in endourology, providing a safer and more efficient approach to managing complex renal stones. By ensuring stable IRP, this technology addresses key limitations of traditional methods and enhances procedural outcomes. Continued research and innovation will be essential to fully realize the potential of AI in improving endourological care.

## Grant information

The authors declare that no grants were involved in supporting this work.

## CRediT authorship contribution statement

**Armand Achmadsyah:** Conceptualization, Data curation, Formal analysis, Investigation, Methodology, Visualization, Writing – original draft, Writing – review & editing. **Favian Ariiq Rahmat:** Conceptualization, Data curation, Methodology, Visualization, Writing – original draft, Writing – review & editing. **Nadhif Faza Ananda:** Conceptualization, Data curation, Methodology, Visualization, Writing – original draft. **Mukhlis Akmal Taher:** Conceptualization, Data curation, Methodology, Visualization, Writing – original draft. **Gerhard Reinaldi Situmorang:** Conceptualization, Formal analysis, Investigation, Resources, Software, Validation, Writing – review & editing. **Widi Atmoko:** Conceptualization, Data curation, Investigation, Resources, Supervision, Validation, Writing – review & editing. **Nur Rasyid:** Conceptualization, Data curation, Formal analysis, Software, Supervision, Validation, Writing – review & editing. **Ponco Birowo:** Conceptualization, Data curation, Formal analysis, Funding acquisition, Investigation, Methodology, Project administration, Resources, Supervision, Validation, Writing – original draft, Writing – review & editing.

## Consent

Written informed consent for the publication of this case report and any associated images was obtained from the patient.

## Ethical approval

Ethical approval was obtained from the Ethics Committee of the Faculty of Medicine, Universitas Indonesia – Cipto Mangunkusumo Hospital (**Approval No.: KET-468/UN2.F1/ETIK/PPM.February 00, 2025**).

## Competing interests

The authors declare that there are no competing interests.

## Data Availability

All data underlying the results are available as part of the article and no additional source data are required.
